# Biocompatibility of magnesium implants in primary human reaming debris-derived cells stem cells in vitro

**DOI:** 10.1007/s10195-015-0364-9

**Published:** 2015-07-08

**Authors:** Olga Charyeva, Olga Dakischew, Ursula Sommer, Christian Heiss, Reinhard Schnettler, Katrin Susanne Lips

**Affiliations:** aap Biomaterials GmbH, Lagerstrasse 11-15, 64807 Dieburg, Germany; Laboratory for Experimental Trauma Surgery, Justus-Liebig University Giessen, Schubertstrasse 81, 35392 Giessen, Germany; Department of Trauma Surgery, University Hospital of Giessen-Marburg, Rudolf-Buchheim-Str. 9, 35385 Giessen, Germany

**Keywords:** Biocompatibility, Magnesium, Human reaming debris-derived cells

## Abstract

**Background:**

Use of magnesium for resorbable metal implants is a new concept in orthopaedic and dental medicine. The majority of studies on magnesium’s biocompatibility in vitro have assessed the short-term effect of magnesium extract on cells. The aim of this study was to evaluate the influence of direct exposure to magnesium alloys on the bioactivity of primary human reaming debris-derived (HRD) cells.

**Materials and methods:**

Pure Mg, Mg2Ag, WE43 and Mg10Gd were tested for biocompatibility. The study consisted of assessment of cell viability by 3-(4,5-dimethylthiazol-2-yl)-2,5-diphenyltetrazolium bromide (MTT) test, evaluation of alkaline phosphatase (ALP) content, and study of cell morphology under light microscopy, scanning electron microscopy (SEM) and transmission electron microscopy (TEM), along with determination of calcification and pH changes induced by magnesium.

**Results:**

The number of viable cells in the presence of Mg2Ag was high over the entire observation period. Inhibition of ALP content in osteogenic differentiating HRD was caused by pure Mg at day 14 and 28. All other magnesium alloys did not affect the ALP content. Exposure of HRD to magnesium increased the amount of lysosomes and endocytotic vesicles. Cellular attachment was generally the best for those crystals that formed on the surface of all materials. A decrease was observed in Ca^2+^ in the medium from day 1 to day 14.

**Conclusions:**

In terms of cell morphology, cell viability and differentiation, cell density and the effect on the surrounding pH, Mg2Ag showed the most promising results. All magnesium materials induced calcification, which is beneficial for orthopaedic and dental applications.

## Introduction

Temporary metal implants that would resorb after bone healing has completed would minimise secondary surgery for implant removal and thus decrease postoperative infections [1–3]. This in turn would decrease high costs related to repeated surgery, reduce recovery periods and thus promote higher quality of life for patients.

A perfect resorbable metal implant should provide enough strength to the healing bone; it should resorb after a set time period, preferably no earlier than 12 weeks [4], and should be non-toxic and cause no damage to the body. Magnesium is a suitable material for biodegradable metal implants because it is biocompatible [5] and does not cause toxicological tissue response. Magnesium occurs naturally in humans since our organism contains about 25 g of this element, about 50–60 % of which is found in bone [6]. Magnesium has been shown to stimulate bone formation, since its ions enhance cell attachment and proliferation [7]. In previous studies, new bone was seen forming in direct contact with magnesium [8, 9]. Finally, magnesium’s mechanical properties are closer to those of healthy bone than titanium’s [10].

However, the problem with magnesium is the formation of hydrogen gas on contact with fluids [4]. In water, magnesium hydroxide accumulates on the surface of the magnesium implant to form a corrosion layer, also known as a degradation layer. While this film slows corrosion under aqueous conditions, it reacts with chlorine ions present in blood to produce highly soluble MgCl_2_ and hydrogen gas [4]. Hydrogen gas is undesirable as it interferes with normal tissue healing and affects primary implant stability in bone.

At the same time, it has been shown that magnesium facilitates calcification and formation of calcium phosphates [11]. Calcium is needed by the body to ensure that bone laid down by osteoblasts is normally mineralised [12]. It has been shown in previous studies that magnesium increases the pH and that high pH promotes Ca^2+^ binding [13, 14]. This is especially important for implants that are to be used for bone fractures.

The majority of studies on biocompatibility of magnesium in vitro have assessed the short-term effects of magnesium extract on cells [15–17]. The aim of this study was to evaluate the influence of magnesium alloys on the bioactivity of primary human reaming debris-derived (HRD) cells with up to 21 days of direct exposure. This, in our opinion, will mimic in vivo conditions more closely.

## Materials and methods

### Sample production

The following materials were used to produce alloys for this study: magnesium (99.99 %, Xinxiang Jiuli Magnesium Co. Ltd., China), yttrium (99.95 % Y, Grirem Advanced Materials Co. Ltd., China), gadolinium (99.95 % Gd, Grirem Advanced Materials Co. Ltd., China), rare-earth mixture (Grirem Advanced Materials Co. Ltd., China) and silver (99.99 % Ag, ESG Edelmetall-Handel GmbH & Co. KG, Germany).

Three magnesium-based materials were produced: Mg2Ag (1.89 % Ag, the rest was Mg), Mg10Gd (8.4 % Gd, the rest was Mg) and WE43 (3.45 % Y, 2.03 % Nd, 0.84 % Ce, the rest was Mg). Pure magnesium (99.99 % Mg) was used as control. The concentrations of magnesium (Mg), Y, neodymium (Nd) and cerium (Ce) were determined by spark emission spectrometer (Spectrolab M, Spektro, Germany), and the concentrations of Ag and Gd were determined by X-ray fluorescence spectrometer (Bruker AXS S4 Explorer, Bruker AXS GmbH, Germany). The materials were cast at Helmholtz-Zentrum Geesthacht Magnesium Innovation Center (HZG-MagIC).

The three magnesium alloys (Mg2Ag, Mg10Gd and WE43) were produced by permanent mould gravity casting. After melting pure Mg, the melt was held at 720 °C and the preheated alloying elements were added with continuous stirring for 15 min. The melt was poured into a preheated (550 °C) permanent steel mould treated with boron nitride. During the casting process, cover gas was used (SF_6_ and Ar mixture). The alloys were homogenised with a T4 heat treatment prior to extrusion in Ar atmosphere at 550 °C (Mg10Gd and WE43) or 420 °C (Mg2Ag) for 6 h. Afterwards, the alloys were extruded indirectly with extrusion ratio of 4:25. The chamber of the extrusion machine was set to 370 °C, and the 30-mm-diameter billets were preheated for 1 h at 370 °C (Mg2Ag), 390 °C (WE43) or 430 °C (Mg10Gd). The extrusion speed was between 3 and 4.5 mm/s. Pure Mg was cast by permanent mould direct chill casting.^27^ The cast billet (*d* = 110 mm) was extruded indirectly with an extrusion ratio of 1:84. The billet temperature was 340 °C, and the speed of the extrusion was 0.7 mm/s. Discs (10 mm diameter, 1.5 mm thickness) were machined from the extruded bars.

### Sample sterilisation and pre-incubation

The samples were sonicated for 20 min in dry isopropanol, dried and gamma-sterilised at the BBF Sterilisation Service GmbH facility (Kernen, Germany) with total dose of 29 kGy. Before seeding the cell culture, the samples were pre-incubated to form a protective degradation layer. Per 0.2 g of sample, 3 mL of F12 K (Gibco^®^, Life Technologies, USA) was used. The samples were kept at 37 °C in 5 % CO_2_ atmosphere for 12 h.

### Isolation of human reaming debris-derived (HRD) cells

HRD cells were cultured from various patients, with the approval of the local Ethics Commission, as described by Wenisch et al. [18]. The adult patients were of different genders and ages and did not display any disease related to bone metabolism. In total, cells from six different patients were taken for this study.

The reaming debris was cultured in Petri dishes with F12 K medium including 20 % foetal calf serum (FCS), 100 U/ml penicillin and 100 μg/g streptomycin. After 4–7 days, HRD cells started to grow out of the debris. When the cells reached confluence after 2–3 weeks, they were trypsinised and transferred to cell culture flasks. All cells were kept at 37 °C in 5 % CO_2_ atmosphere.

### Cell viability

To determine cell viability, an MTT assay was conducted according to Mosmann et al. [19]. Briefly, 10,000 cells/cm^2^ were seeded into 12-well plates containing pre-incubated magnesium discs and F12 K medium with 20 % FCS and 100 μg/g streptomycin. Controls with no magnesium discs but only HRDs were used as well. The cell medium was changed every second day during the experiment. Duplicates were used for each material and patient, resulting in testing of 12 wells per specimen. After 24 h, 7 and 21 days, MTT solution was added to cell medium. The cells were then incubated in the dark for 4 h at 37 °C. Subsequently, the cell medium was discarded, and the cells were lysed with 0.004 N HCl in isopropanol. The cell lysates were centrifuged, and supernatants were transferred as triplets to a 96-well plate. Adsorption was measured at 570 and 630 nm using a Synergy HT microplate reader (BioTek, Bad Friedrichshall, Germany). The MTT assay was also performed for magnesium discs not seeded with cell culture to exclude the material’s effect on the test and see only how the cells reacted during the assay.

Additionally, cell morphology was studied by inverted light microscopy using a Leica microscope type 090-135.002 (Leica Microsystems GmbH, Wetzlar, Germany) equipped with a Nikon Ds-Fi1 digital camera (Nikon, Düsseldorf, Germany).

### Alkaline phosphatase (ALP) content

As an indicator of changes in the differentiation behaviour of the bone-forming cells caused by the test substances, a SensoLyte^®^ pNPP alkaline phosphatase assay (AnaSpec, Fremont, CA) was applied after 24 h and 7, 14, 21 and 28 days of culturing in Dulbecco’s modified Eagle’s medium (DMEM), low glucose with l-glutamine, 10 % FCS, 100 U/ml penicillin, 100 μg/g streptomycin, 0.1 μM dexamethasone, 0.005 μM ascorbic acid and 10 mM β-glycerol phosphate to induce osteogenic differentiation. The cell medium was changed every second day during the experiment. Duplicates were used for each material and patient, for a total of 12 wells per specimen. Controls with no magnesium discs but only HRDs were used as well.

The cells were washed and frozen at −80 °C. After thawing, the cell number was measured using a PicoGreen^®^ dsDNA quantitation assay (Invitrogen, Eugene, OR) according to the manufacturer’s protocol. Cells were lysed with 1 % Triton X-100 in phosphate-buffered saline. The cell lysates were centrifuged, and the supernatants were mixed with PicoGreen^®^ working solution in a 96-well plate. The samples were excited at 485 nm, and the fluorescence emission intensity was measured at 528 nm. The cells that were lysed for the PicoGreen^®^ assay were centrifuged, and the supernatants were diluted in a specific assay buffer. ALP substrate was applied to the diluted samples, and the absorbance was measured at 405 nm. The absolute amounts of ALP were correlated with the cell numbers obtained from the PicoGreen^®^ assay. Both ALP and PicoGreen^®^ assays were additionally performed for magnesium discs not seeded with cell culture to exclude the material’s effect on the test and see only how the cells reacted during the assays.

### Transmission electron microscopy

HRD cells seeded in chamber slides (Nalge Nunc International, Rochester, NY) were incubated with and without magnesium discs for 21 days. The cell layer was fixed for 30 min with 2 % paraformaldehyde in 0.1 M sodium phosphate buffer (pH 7.2–7.4) with 2 % glutaraldehyde and 0.02 % picric acid, followed by 20 min fixation with 1 % osmium tetroxide in 0.1 M sodium cacodylate buffer (pH 7.2–7.4). The samples were dehydrated and embedded in Epon before ultrathin sections (80–100 nm) were applied to collodion-coated copper grids. Analysis was done with a LEO 912 transmission electron microscope (Carl Zeiss AG, Oberkochen, Germany) at 80 kV accelerating voltage, equipped with a TRS SharpEye slow-scan dual-speed charge-coupled device (CCD) camera (Albert Troendle Prototypentwicklung, Moorenweis, Germany).

### Scanning electron microscopy

HRD cells were cultivated on magnesium discs for 7 and 21 days. Controls with no magnesium discs but only HRDs were used as well. Subsequently, the cells were fixed in 2 % glutaraldehyde in 0.1 M Na-phosphate buffer (pH 7.4) for 1 h at room temperature, followed by dehydration in a graded series of ethanol and critical-point drying. The specimens were mounted together on aluminium pin stubs with the help of adhesive carbon pads. The specimens were then sputter-coated with gold/palladium (SC7640 sputter coater, VG Microtech, Uckfield, East Sussex, UK) and assessed using a LEO 1530 (LEO Elektronenmikroskopie GmbH, Oberkochen, Germany) field-emission scanning electron microscope operated at 7.5 or 15 kV.

### Determination of Ca^2+^ consumption and pH

At established time points, medium was collected and analysed for Ca^2+^ in solution and pH. The concentration of Ca^2+^ was measured using a calcium analyser (9180 electrolyte analyzer, Roche, Mannheim, Germany), and pH measurements were carried out using a pH-meter (Titan X, Fisher Scientific GmbH, Schwerte, Germany) for each time point. The control group for this investigation consisted of well plates that contained only HRDs and medium but no magnesium.

### Statistical analysis

Data were analysed using the Statistical Package for the Social Sciences (SPSS, v18, SPSS Inc., Chicago, USA). The significance level was set at 5 %. The treatment groups were compared with the control group without magnesium by Mann–Whitney *U*-test. The results are expressed as means. The graphs were plotted with SPSS.

## Results

### Cell viability

Cell viability values are presented in Table 1. It was observed that Mg2Ag had the highest cell viability of all materials during the whole observation period. Cells with pure Mg had high viability at day 1 (93.4 ± 25.3 %), but then the viability decreased and reached 24.0 ± 19.5 % at day 21. The same pattern was observed for WE43 with initial viability of 57.1 ± 14.4 %, reaching 26.3 ± 3.6 % at day 21. Mg10Gd had the lowest cell viability of all tested materials over the entire observation period.

**Table 1:** MTT results for HRDs after exposure to different magnesium materials over time

Material	Viability (% of control) ± SD (%), day 1	Viability (% of control) ± SD (%), day 7	Viability (% of control) ± SD (%), day 21
Pure Mg	93.4 ± 25.3	13.9 ± 5.0	24.0 ± 19.5
WE43	57.1 ± 14.4	30.5 ± 9.2	26.3 ± 3.6
Mg10Gd	37.0 ± 13.1	18.1 ± 7.1	54.7 ± 7.2
Mg2Ag	113.4 ± 29.8	63.3 ± 11.0	98.5 ± 12.0

### Alkaline phosphatase content

**Fig. 1:**
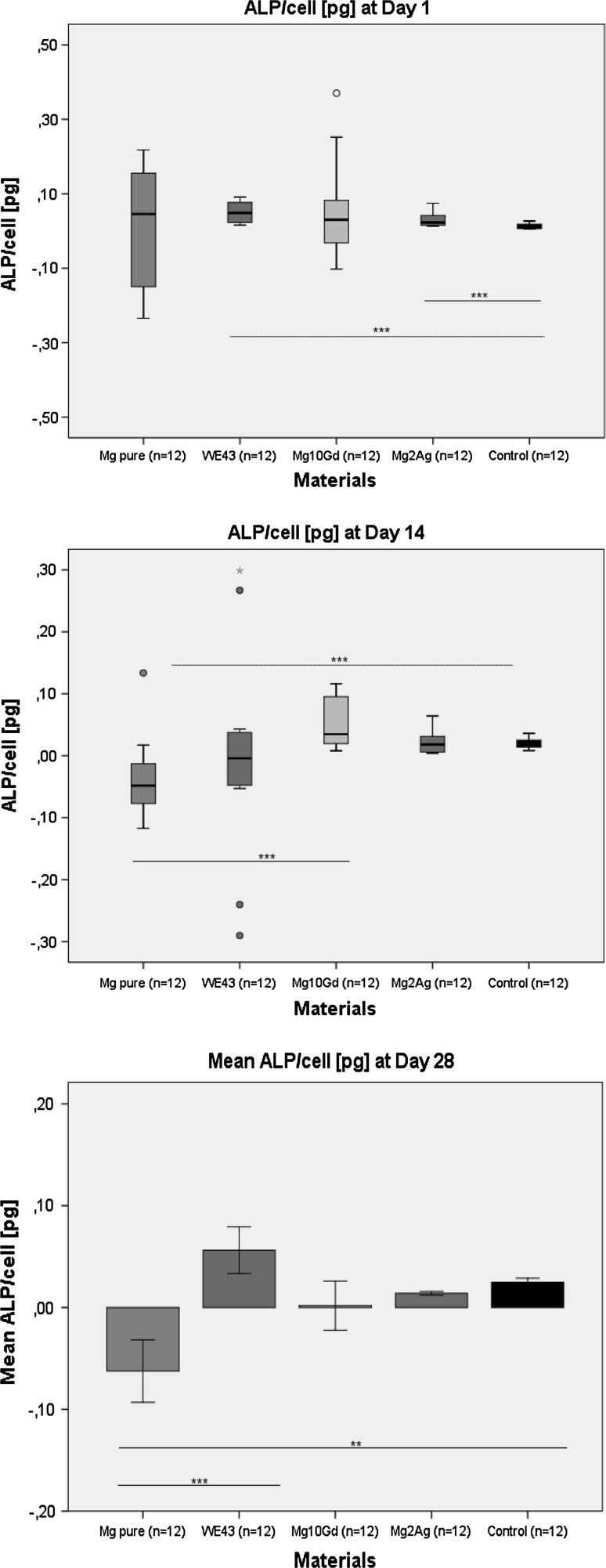
ALP content at day 1, 14 and 28

ALP content is an important factor in bone mineral formation and shows a scale of changes during differentiation. No inhibition of ALP activity caused by Mg2Ag, Mg10Gd and WE43 was observed in osteogenic differentiating HRD at days 14 and 28 (Fig. 1). At day 1, the ALP content was significantly higher for Mg2Ag (*p* = 0.004) and WE43 (*p* = 0.003) compared with control. Significantly low values for ALP content compared with the control group were observed for pure Mg at day 14 (*p* = 0.002) and day 28 (*p* = 0.004).

### Cell morphology

**Fig. 2:**
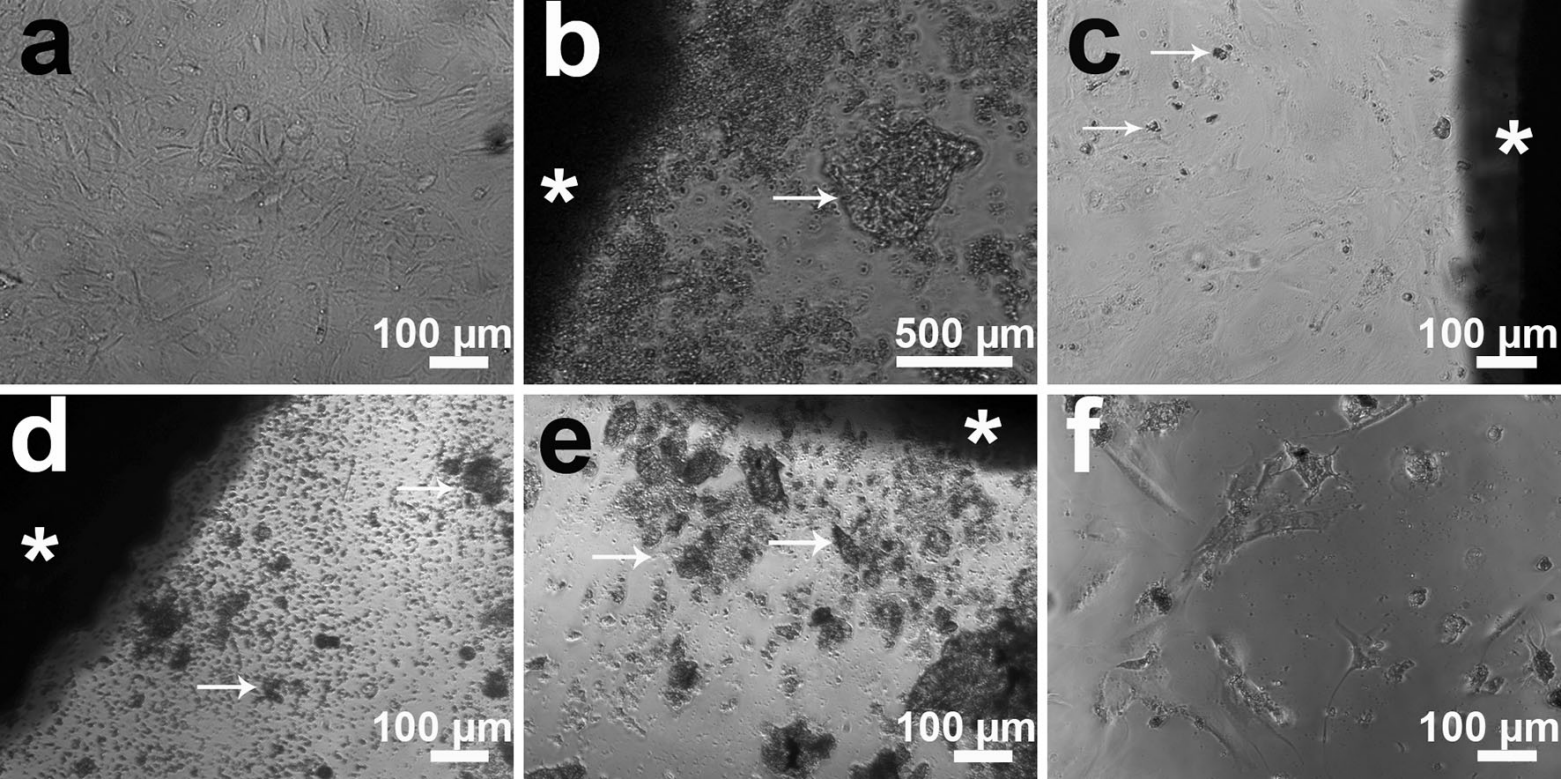
Morphology of HRD at 7 days: **a** control group, the well is densely covered with cells; **b** pure Mg, many fewer cells compared with control; **c** Mg2Ag, the well is densely covered with cells; **d** Mg10Gd, many fewer cells compared with control; **e** WE43, similar in appearance to pure Mg and Mg10Gd with very few cells observed in material’s proximity; **f** for pure Mg, Mg10Gd and WE43, the cells were found mainly at the well edge (the image shows Mg10Gd’s well edge). *Asterisk* magnesium disc, *arrows* products of degradation

Changes in cell morphology were detected by inverted light microscopy for HRD and for osteogenic differentiating HRD. After 7 days of exposure to pure Mg, Mg10Gd and WE43, HRD showed a reduction in cell number and an increasing amount of cell debris in the medium (Fig. 2). Few or no cells were seen in direct contact with pure Mg, Mg10Gd and WE43. Instead, the cells were found on the edge of the wells (Fig. 2f). The reduction in cell number was more apparent for pure Mg than in any other group. The cell morphology in the presence of Mg2Ag was similar to control, and the cells were directly contacting the Mg2Ag discs (Fig. 2c).

**Fig. 3:**
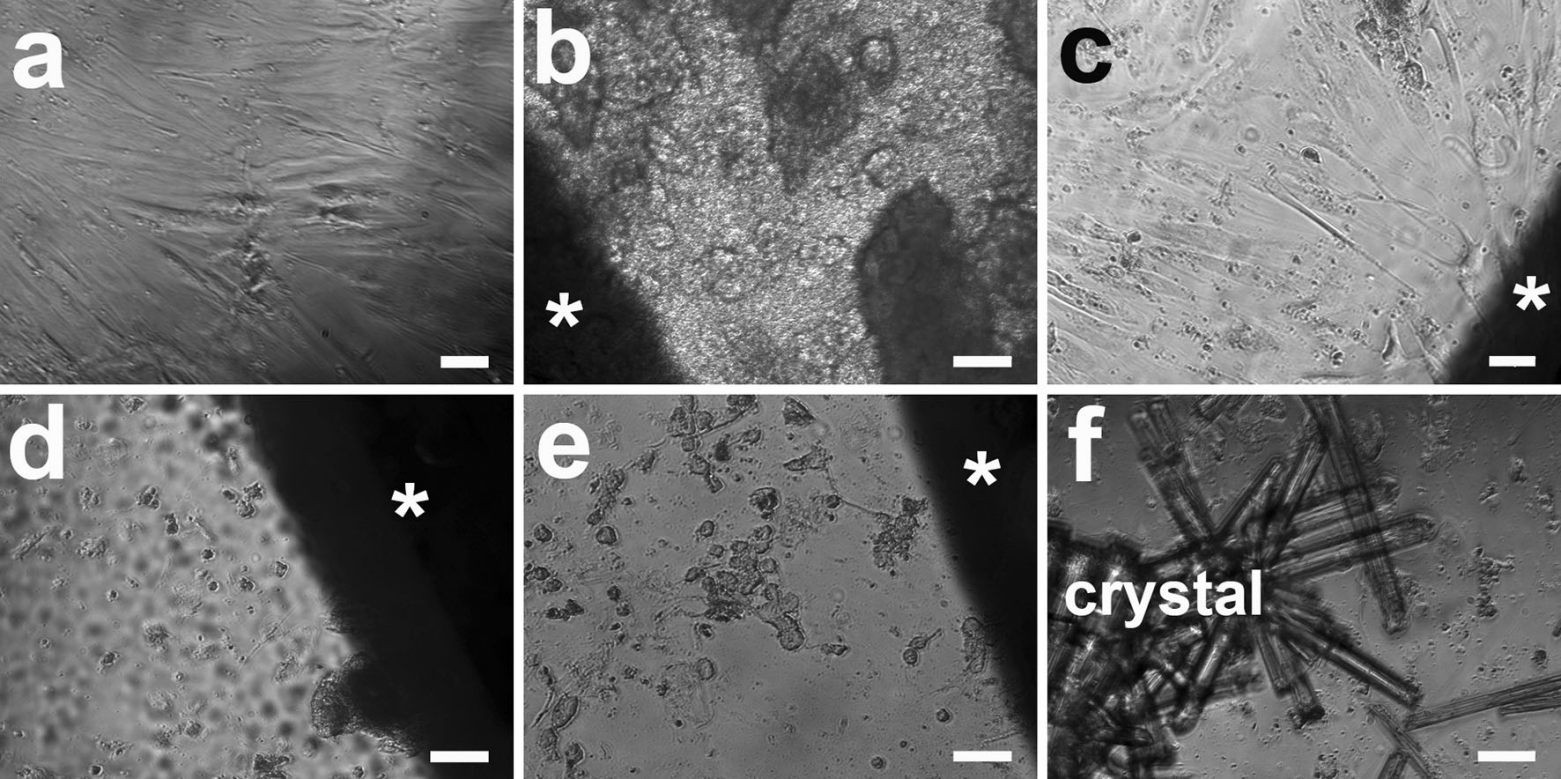
Morphology of HRD at 21 days: **a** control group, the well is densely covered with cells; **b** pure Mg, almost no cells compared with control; **c** Mg2Ag, the well is densely covered with cells; **d** Mg10Gd, many fewer cells compared with control but more than at day 7; **e** WE43, similar appearance to Mg10Gd with somewhat more cells than at day 7; **f** crystal formation was observed for all materials (the image shows the pure Mg well). *Asterisk* magnesium disc. *Scale bar* 100 µm

At 21 days, the HRD in WE43 and Mg10Gd started to appear closer to the disc, although their number was still low compared with control or Mg2Ag (Fig. 3). For pure Mg, cells were still only found around the edge of the well and not in proximity to the material. For Mg2Ag, the cell morphology was most similar to the control with high cell density directly contacting the discs.

**Fig. 4:**
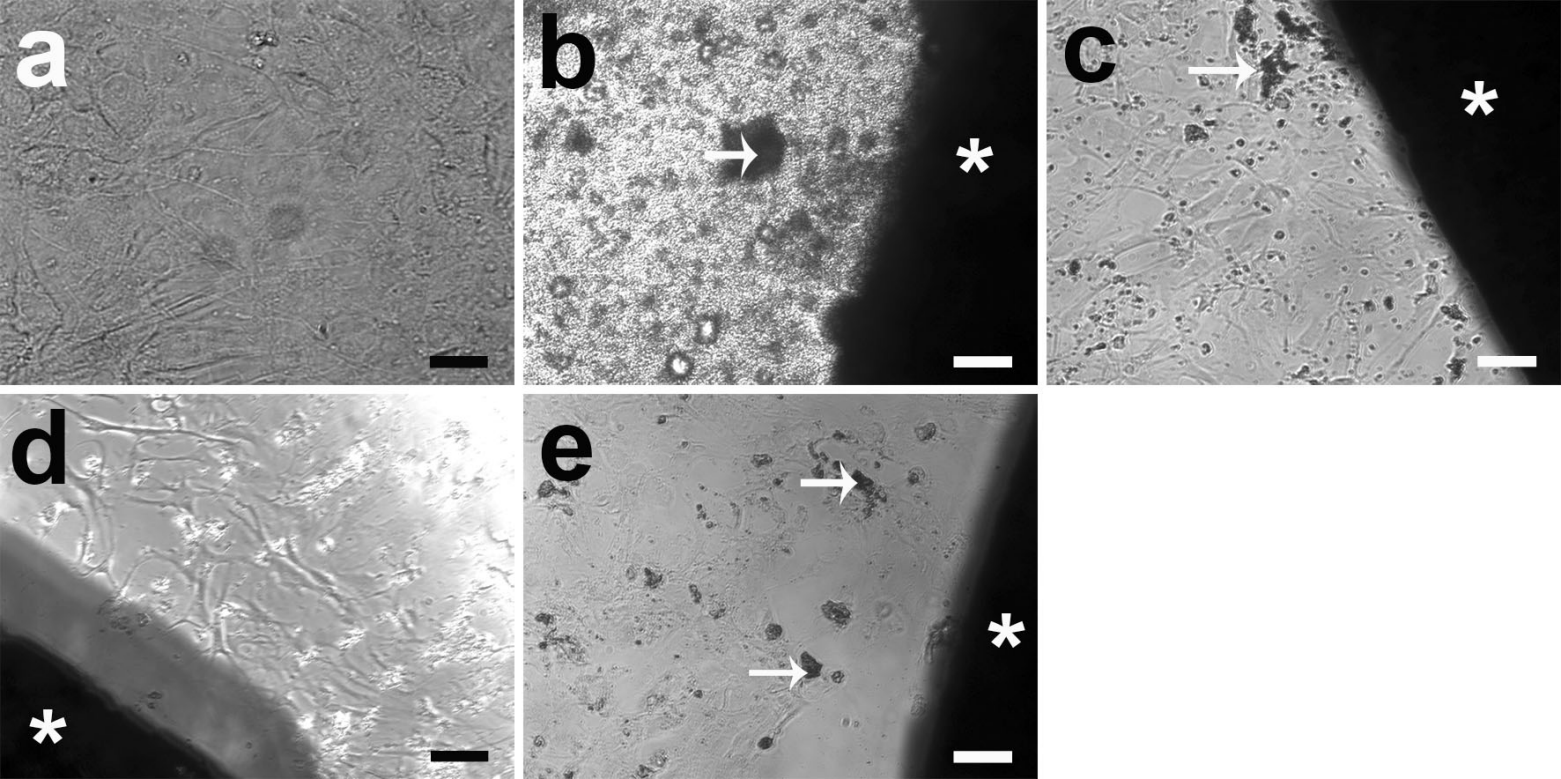
Morphology of osteogenic differentiating HRD at 28 days: **a** control group, the well is densely covered with cells; **b** pure Mg, few cells compared with control; **c** Mg2Ag, the well is densely covered with cells; **d** Mg10Gd, the well is densely covered with cells; **e** WE43, the well is densely covered with cells. *Asterisk* magnesium disc, *arrows* products of degradation. *Scale bar* 100 µm

The osteogenic differentiating HRD showed a similar pattern of cell morphology and cell number as HRD (Fig. 4). No cells were found in direct contact with pure Mg (Fig. 4b). Mg2Ag was the most similar to control at all of the time points regarding morphology and cell density. At day 28, the osteogenic differentiating HRD in the Mg2Ag group were still most similar to the control regarding cell number (Fig. 4c). More cells appeared around Mg10Gd and WE43 at day 28 compared with other time points for these materials (Fig. 4d, e).

### Transmission electron microscopy

**Fig. 5:**
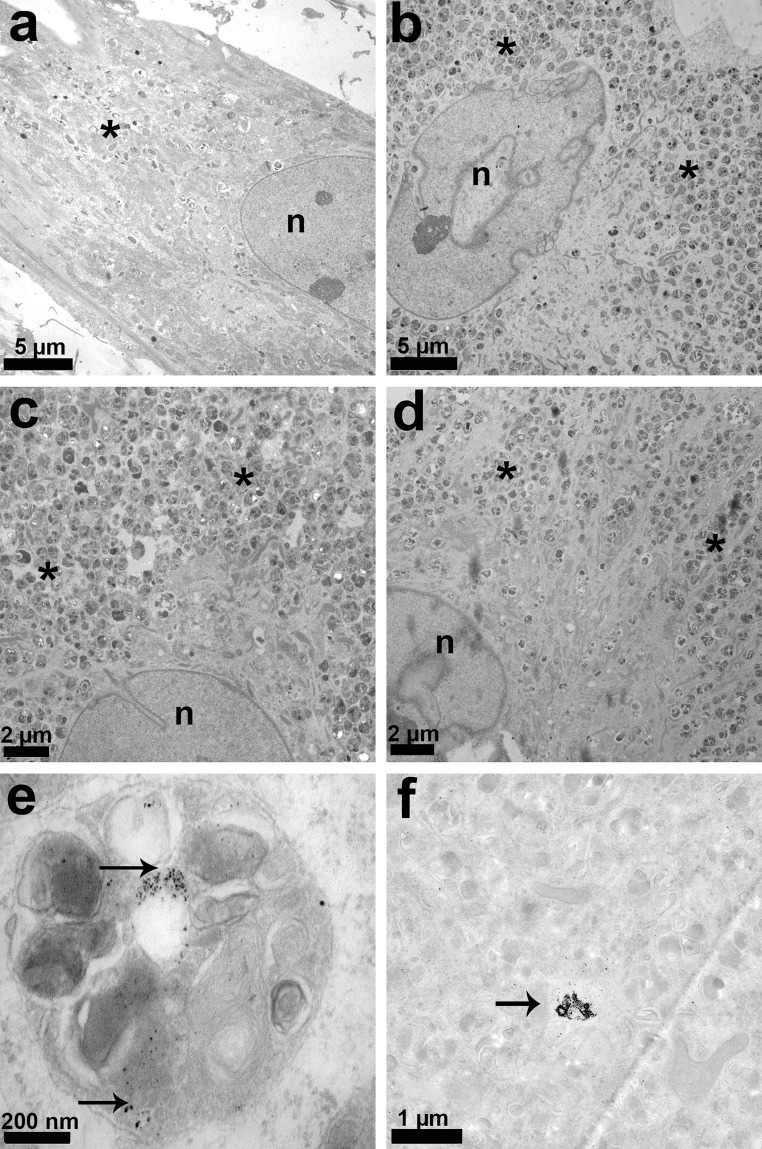
TEM analysis at day 21: **a** control, **b** Mg2Ag, **c** Mg10Gd, **d** WE43, **e** lysosome of HRD cultured with Mg2Ag (note the degradation particles, *arrows*); **f** cytoplasm of HRD cultured with Mg2Ag (note the degradation particles, *arrows*). *Asterisk* lysosomes, endocytotic vesicles, *n* nucleus. Note the high amount of lysosomes and endocytotic vesicles in **b**–**d**

Intracellular structure was examined after exposure of HRD to magnesium samples for 21 days. It was observed that the number of lysosomes and endocytotic vesicles was higher in the HRD exposed to magnesium alloys than in the control (Fig. 5). In Mg2Ag, degraded material particles were found inside the lysosomes (Fig. 5e) and in the cytoplasm (Fig. 5f). Degradation particles were not observed in other groups.

### Scanning electron microscopy

**Fig. 6:**
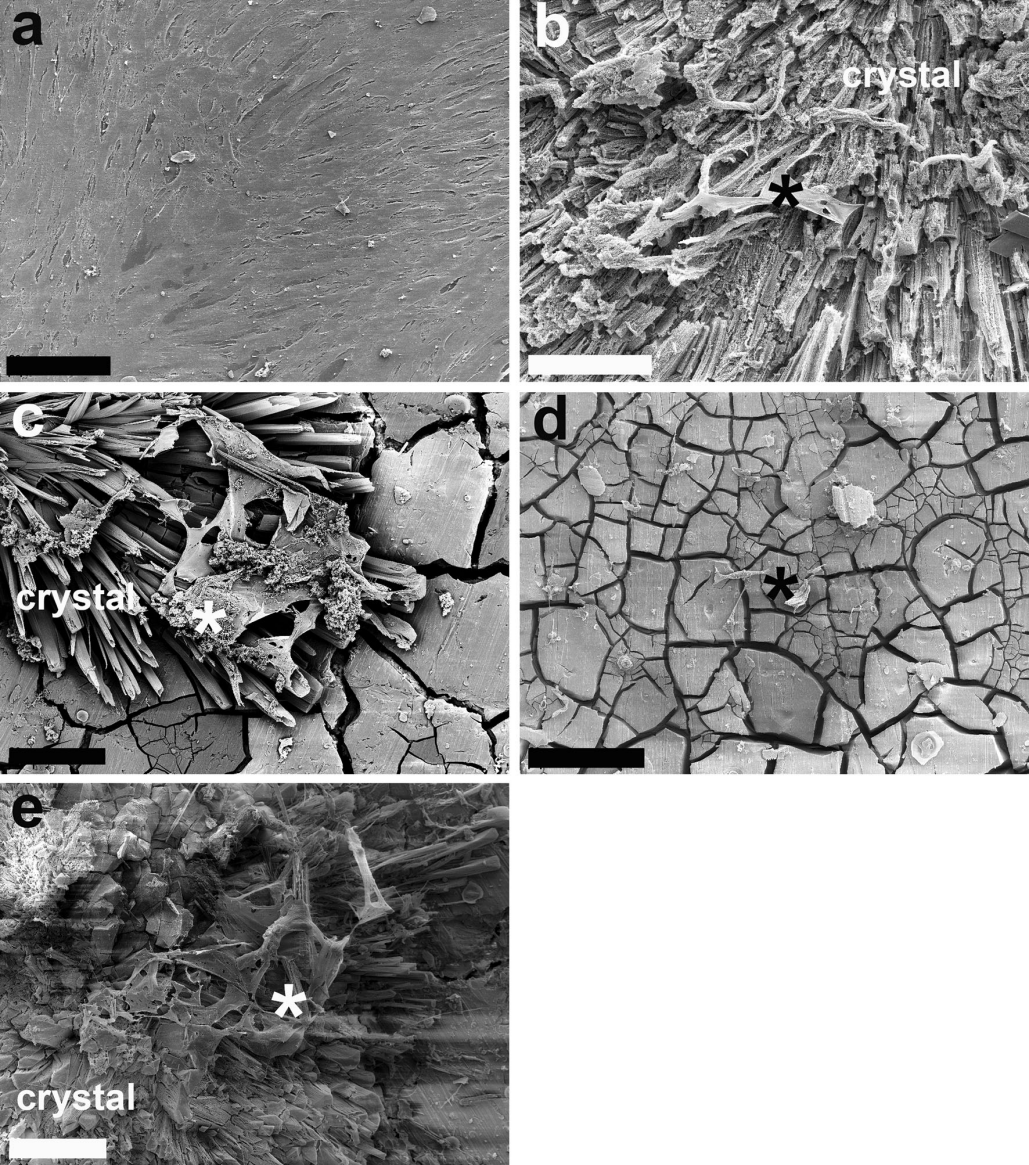
SEM analysis at day 7: **a** control, densely covered with HRDs (*scale bar* 100 µm); **b** pure Mg, **c** Mg2Ag, **d** Mg10Gd, **e** WE43. Note crystal formation on material surface in **b**–**e**, and cell attachment to the crystal compared with the smooth surface in **d**. Cells are marked with an *asterisk*. *Scale bar* in **b**–**e** 5 µm

Cellular attachment to magnesium specimens was studied under SEM after incubating HRD with magnesium for 7 and 21 days. It was observed that the cells attached to the degradation layer and to the crystals forming on the surface of magnesium (Fig. 6). Cell pseudopodia were numerous whenever crystals formed on material surfaces (Fig. 6b, c, e). Few pseudopodia were seen on smoother surfaces (Fig. 6d). No difference between 7 and 21 days was observed regarding the number of attached cells or their morphology.

### Ca^2+^ consumption

**Fig. 7:**
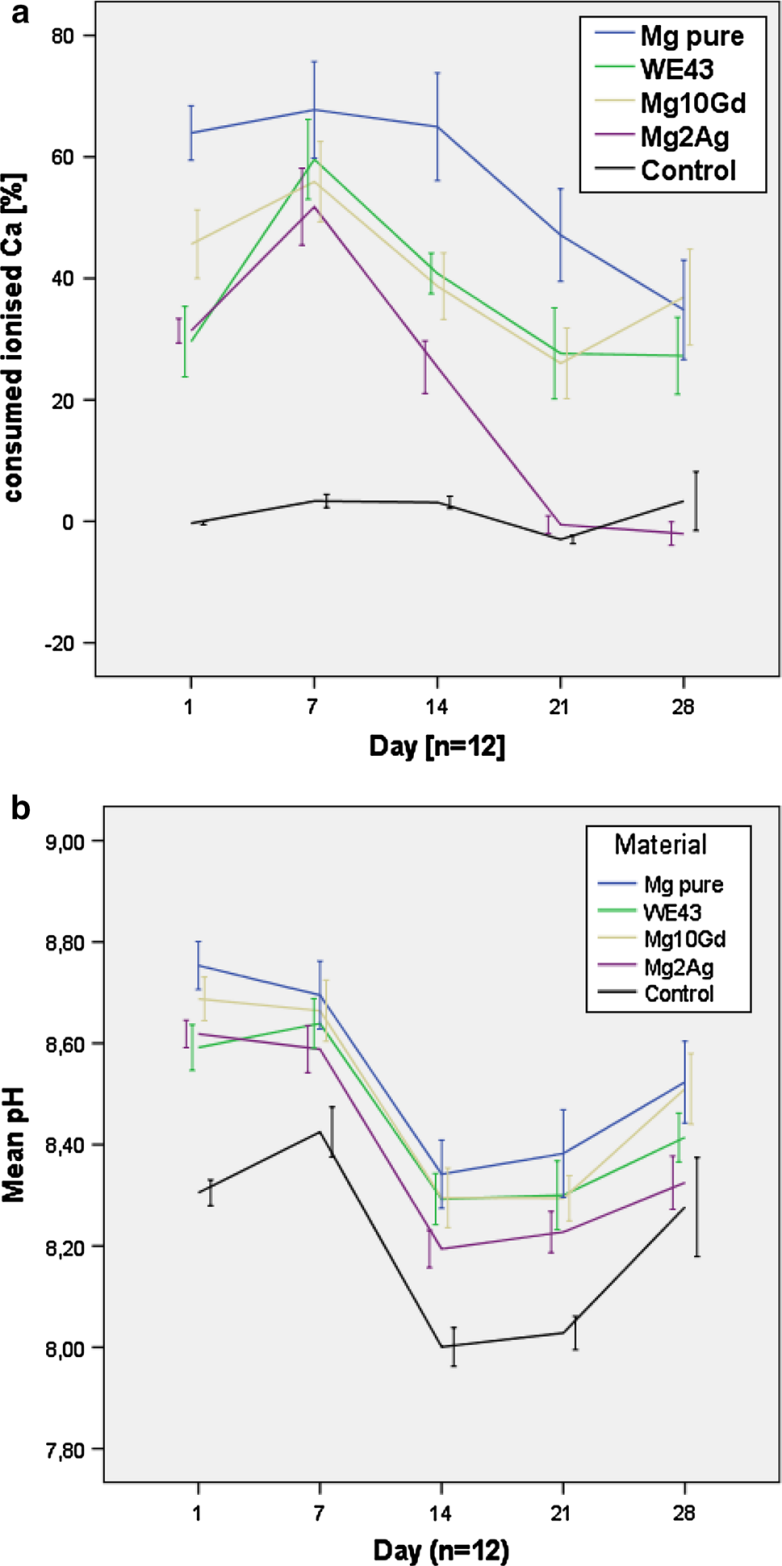
**a** Consumed ionised calcium at different observation points. **b** pH over time

Calcification induced by magnesium specimens was studied by measuring Ca^2+^ consumption from the surrounding medium. A decrease in free calcium ions was observed in the medium around all magnesium materials from day 1 to day 14. Between day 14 and day 21, Ca^2+^ was released into the medium. Between day 21 and 28, Ca^2+^ levels were stable for pure Mg, WE43 and Mg10Gd but decreased for Mg2Ag (Fig. 7a). Ca^2+^ consumption levels were stable in the control over the whole study period. The values for Mg2Ag were most similar to those of the control out of all groups starting on day 21.

### pH measurements

It was seen that all magnesium materials increased the pH of the medium compared with the control group. The following general pattern was observed for all groups: the pH values were stable up to day 7, a sudden pH drop occurred on day 14, then the pH tended to increase slightly up to day 28. The pH values for Mg2Ag were most similar to the control. Pure Mg caused the greatest increase in pH among all groups, and this increase was statistically significant compared with Mg2Ag (*p* ≤ 0.003) but not compared with Mg10Gd (*p* ≥ 0.02) or WE43 (*p* ≥ 0.02). No correlation between pH and Ca^2+^ consumption was found in this study.

## Discussion

This study looked at the direct long-term effect of magnesium alloys on primary HRDs. Cell viability, differentiation and morphology as well as pH and calcium uptake were analysed to assess the overall biocompatibility of the tested materials. We evaluated the long-term effects of magnesium on human cells to simulate the in vivo situation as closely as possible.

Mg2Ag had high cell viability from day 1 (113.4 ± 29.8 %) to day 21 (98.5 ± 12.0 %). It was also shown in previous works [15] that Mg-Ag alloys have negligible cytotoxicity and sound cytocompatibility. Pure Mg had high viability at the very first day (93.4 ± 25.3 %), but then the viability decreased to 24.0 ± 19.5 % at day 21. Mg10Gd and WE43 impaired cell viability in this study. Previous studies have shown higher values for cell viability measured by MTT test compared with the present study [15–17]. The difference between this work and previous publications is that the present study applied the longest in vitro incubation times for magnesium alloys tested up to now. The HRDs were kept in direct contact with the magnesium samples and not in magnesium extract, as done in most studies [15–17].

An important drawback of tetrazolium-based tests is that the difference between cytotoxic (cell death) and cytostatic (reduced growth rate) effects cannot be distinguished [20]. We thus looked at cell morphology under light microscopy, TEM and SEM.

After examination under SEM and light microscopy, it was revealed that the number of cells decreased in the presence of pure Mg, Mg10Gd and WE43. These materials seem to have long-term cytotoxic effects on HRD when placed in direct contact with the cells. This explains the low viability values.

The cell number was high and the cells had normal morphology in Mg2Ag groups. However, the cell viability was lowest at day 7 (63.3 ± 11.0 %) for Mg2Ag. TEM analysis revealed an elevated amount of lysosomes which contained degraded magnesium particles. Degradation particles were also found in the cytoplasm. The presence of high amounts of degradation products inside the HRDs could explain the lower cell viability values for Mg2Ag at day 7. It was shown in previous studies that uptake of material particles leads to induction of cell stress which triggers cytotoxicity [21].

ALP activity in HRDs is an important factor in bone mineral formation and shows a range of changes during differentiation. Inhibition of ALP activity in osteogenic differentiating HRD was caused by pure Mg at day 14 and 28. All other magnesium alloys did not affect the ALP activity. In this respect, our study shows similar results to previous research in this area [22], despite the fact that we observed osteogenic differentiation over much longer periods and using direct contact of cells with magnesium.

SEM analysis revealed that the cellular attachment was generally best to crystals generated by degradation products on the material surface. Crystals have been seen forming on magnesium alloys such as Mg-Ag in previous studies [15]. Formation of calcium phosphates [Ca_*x*_(PO_4_)_*x*_] was also observed in previous publications [11]. Interestingly, the crystal distribution was not homogeneous throughout the corrosion layer. In this sense, our results are similar to earlier findings [11, 15].

The fact that the cells attached to the crystalline structures more readily than to the overall material surface and developed numerous pseudopodia can be explained by the rough structure of crystals, and by the chemical composition of these crystals. It was previously shown that cells attach better to certain surfaces with preferable average surface roughness of ~0.5 μm up to ~8.5 μm [23]. Values below or above this range diminish the cells’ ability to bind to the surface.

The chemical composition of the crystals and the degradation layer formed on the magnesium’s surface can also explain the better attachment of the cells to these structures. Their chemical composition consists of calcium, phosphorus, magnesium and oxygen [14]. Thus, the cells attach to already reacted material where they are not mechanically disturbed by hydrogen gas produced as a by-product of degradation. The formation of the degradation layer could also explain the increase in cell density around Mg10Gd and WE43 after 21 days of incubation.

Based on previous findings, the following model for the formation of a corrosion layer has been suggested [14]: (1) initial metal corrosion based on contact with water molecules leads to release of Mg ions, and a thin Mg(OH)_2_ and MgCO_3_ layer is formed; (2) The corrosion slows down, and a second layer consisting of amino acids and organic matter is formed; (3) Both layers together shield the sensitive environment around the material and enable cells to grow on the material [14]. Such a complex process of corrosion layer formation could explain the cell viability increase at day 21 for Mg2Ag, Pure Mg and Mg10Gd observed in our experiments.

All magnesium-based materials decreased the amount of Ca^2+^ in this study. As shown in previous studies, Mg^2+^ promotes formation of calcium phosphates and consequently decreases the amount of free Ca^2+^ ions in the medium [11, 14]. In this regard, our results are consistent with earlier works. Sufficient supply of calcium is vital to ensure that bone laid down by osteoblasts is normally mineralised [12]. Calcification is thus advantageous for bone implants that are to be used in the orthopaedic and maxillofacial fields.

It was shown in previous studies that magnesium increases the pH and that high pH promotes Ca^2+^ binding [13, 14]. In this study, it was also revealed that pH shifts to alkaline values in the presence of magnesium, but to somewhat different degrees for the different alloys. However, no statistical correlation was observed between pH and consumed Ca^2+^.

In conclusion, our study reveals the long-term effects of magnesium materials on human HRDs seeded directly onto magnesium discs. In respect to cell morphology, cell density and the effect on the surrounding pH, Mg2Ag showed the most promising results. However, the mechanism of cell stress induction and cytotoxicity needs to be further studied to enable prediction of possible health risks.
